# Assessment of salivary cadmium levels and breast density in the Marin Women's Study

**DOI:** 10.1002/cam4.6973

**Published:** 2024-02-01

**Authors:** Michaela F. George, Shayne Paff, Jenyse Rojo, Mark Powell, Christopher Benz, Karl Pope, Karla Kerlikowske, John Shepard, Matthew Willis, Rochelle Ereman, LeeAnn Prebil

**Affiliations:** ^1^ Global Public Health Department, School of Health and Natural Sciences Dominican University of California San Rafael California USA; ^2^ Epidemiology and Community Health Marin County Department of Health and Human Services San Rafael California USA; ^3^ Zero Breast Cancer Buck Institute for Research on Aging San Rafael California USA; ^4^ Cancer & Developmental Therapeutics Buck Institute for Research on Aging San Rafael California USA; ^5^ Department of Medicine, Epidemiology and Biostatistics University of California San Francisco California USA; ^6^ Leonard Davis School of Gerontology University of Southern California Los Angeles California USA; ^7^ Population Sciences in the Pacific Program, Cancer Epidemiology University of Hawaii Cancer Center Honolulu Hawaii USA

**Keywords:** biomarkers, breast cancer, cancer prevention, cancer risk factors, community outreach, epidemiology

## Abstract

**Background:**

We aimed to determine if salivary cadmium (Cd) levels had any association with breast density, hoping to establish a less invasive cost‐effective method of stratifying Cd burden as an environmental breast cancer risk factor.

**Methods:**

Salivary Cd levels were quantified from the Marin Women's Study, a Marin County, California population composite. Volumetric compositional breast density (BD_sxa_) data were measured by single x‐ray absorptiometry techniques. Digital screening mammography was performed by the San Francisco Mammography Registry. Radiologists reviewed mammograms and assigned a Breast Imaging‐Reporting and Data System score. Early morning salivary Cd samples were assayed. Association analyses were then performed.

**Results:**

Cd was quantifiable in over 90% of saliva samples (mean = 55.7 pg/L, SD = 29). Women with higher saliva Cd levels had a non‐significant odds ratio of 1.34 with BI‐RAD scores (3 or 4) (95% CI 0.75–2.39, *p* = 0.329). Cd levels were higher in current smokers (mean = 61.4 pg/L, SD = 34.8) than former smokers or non‐smokers. These results were non‐significant. Pilot data revealed that higher age and higher BMI were associated with higher BI‐RAD scores (*p* < 0.001).

**Conclusion:**

Salivary Cd is a viable quantification source in large epidemiologic studies. Association analyses between Cd levels and breast density may provide additional information for breast cancer risk assessment, risk reduction plans, and future research directions. Further work is needed to demonstrate a more robust testing protocol before the extent of its usefulness can be established.

## 1 INTRODUCTION

Cadmium (Cd) is classified as a heavy metal carcinogen by the International Agency for Research on Cancer.^1^, ^2^, ^3^, ^4^, ^5^, ^6^, ^7^, ^8^, ^9^ It is environmentally ubiquitous; found in the soil and can become concentrated in tobacco plants, vegetables, and grains.^3^, ^4^, ^5^, ^6^, ^9^, ^10^, ^11^, ^12^, ^13^ Cd is also released into the atmosphere from motor vehicle fuel and is used in the production of batteries, fertilizers, and plastics.^14^, ^15^ In 90%–95% of cases, Cd introduction into the human body occurs mainly through inhalation, while the remaining 5%–10% is through ingestion.^2^, ^3^, ^14^, ^15^, ^16^, ^17^ Tobacco smoke and diet are the main sources of non‐occupational environmental Cd exposure.^3^, ^6^, ^14^, ^17^ Women have been found to have a higher gastrointestinal absorption rate of Cd than men.^18^, ^19^, ^20^

Once absorbed by the body Cd can be stored for decades and interferes with multiple cellular processes, including DNA repair and methylation.^21^, ^22^, ^23^, ^24^ It induces oxidative stress and inflammation, stimulates cellular proliferation, and disrupts tight junctions between cells, theoretically contributing to tumor development.^25^, ^26^, ^27^, ^28^, ^29^, ^30^ Cd exposure had reportedly been associated with female breast cancer in some studies,^31^, ^32^, ^33^ as it acts on estrogenic signaling pathways that may trigger the malignant transformation of breast cancer cells in vitro and in vivo, independent of estrogen receptor (ER)‐α.^31^, ^32^, ^33^, ^34^, ^35^ Thus, Cd is a high concern for hormone‐related breast cancer. Cd concentrations in breast tissue from women diagnosed with breast cancer have been at higher levels compared to concentrations in non‐cancerous breast tissue.^35^, ^36^

Cd levels are most commonly measured through serum and urine sampling, with urinary Cd (U‐Cd) being the current standard diagnostic test^37^, ^38^ to assess for Cd exposure. However, U‐Cd does not seem to reflect chronic low Cd levels but rather is more reflective of recent Cd exposure.^39^ On the contrary, serum Cd measurements continue to show high levels of Cd even after exposure cessation,^39^, ^40^ which indicates that serum Cd levels are more reflective of a previous long‐term exposure rather than recent exposure. Both serum and U‐Cd sampling methods are not the least invasive or convenient. Saliva Cd sampling could provide a noninvasive and convenient method for specimen collection, which may be a more useful biological monitoring tool for large‐scale population screening.^40^, ^41^ Limitations of saliva Cd sampling include variations in the saliva flow rate, possible blood contamination during collection, absence of standard laboratory reference values, and the presence of other metal compounds from dental procedures that may confound results.^41^, ^42^

To our knowledge, little is known about salivary Cd sampling in the context of breast cancer risk factor assessment. In this current pilot feasibility study, we assessed if there was an association between salivary Cd levels and breast density, a known breast cancer risk factor.

## 2 MATERIALS AND METHODS

### 2.1 Study population

Data used for this feasibility study were collected from the Marin Women's Study (MWS), a mammography‐population‐based study of Marin County women residents between 2006 and 2009, which enrolled over 13,000 women.^43^ The mammography imaging diagnostic sites involved in the MWS were part of the San Francisco Mammography Registry (SFMR), which is one of seven registries included in the National Cancer Institute Breast Cancer Surveillance Consortium. An estimated 80% of mammograms performed on Marin residents were conducted within centers associated with these locations. A total of 8700 salivary specimens were received from the enrolled women in the MWS. A subset of 400 saliva samples was assayed for Cd levels (pg/L).

The MWS was developed to create a data repository, linking risk factors to biospecimens and breast density measures to examine the associations between known and suspected risk factors, namely, breast density as one. This study was approved by the Marin General Hospital Institutional Review Board and the Kaiser Permanente Northern California Institutional Review Board. All participants provided informed consent to fully participate in the study.

### 2.2 Questionnaire

Each participant enrolled in the MWS study was asked to complete a 20‐page questionnaire, in collaboration with the Kaiser Foundation, and report in‐depth information on reproductive history, life course socioeconomic status, alcohol use, exogenous hormone use, family history of breast cancer, and other risk factors (Figure S1A). History of smoking was collected using an item that asked respondents if they had smoked at least 100 cigarettes in their lifetime. If so, the ages when they smoked and the average number of daily cigarettes smoked during that period were also investigated. The question of having smoked at least 100 cigarettes was dichotomized into “Never Smoked” and “Ever Smoked” for purposes of the current analysis. Self‐reported height and weight were collected from the SFMR. Data were categorized into overweight/obese (BMI of 25 kg/m^2^ or greater) versus underweight/normal weight (BMI under 25 kg/m^2^). Participants were asked to provide information on average daily/weekly/monthly dietary intake, including average servings of whole grains. Servings were quantified as ½ cup of cereal, crackers, brown rice, pasta, one corn tortilla, or one slice of bread. The whole grain dietary intake variable was dichotomized into “Yes” or “No” to correspond to whether participants had one or more cups of whole grain per day. Vegetable consumption was elicited by asking participants about their usual daily/weekly/monthly servings, with a serving size equal to ½ cup. For vegetable consumption, women between the ages of 19–51+ years need about 2–2½ cups per day. Vegetable intake was dichotomized into “Yes” or “No” to correspond to whether participants usually consumed two or more cups of vegetables per day.

### 2.3 Breast density measures

Data from the MWS were linked to the SFMR to obtain information on breast density, measured as percent fibro‐glandular volume (%FGV), and Breast Imaging‐Reporting and Data System (BI‐RADS) results. The %FGV was assessed by the method of single x‐ray absorptiometry (SXA), which measures the percentage of fibro‐glandular tissue volume. This tool is both accurate and precise.^44^ In comparison to other breast density measures, SXA is less subjective and has absolute reference standards.^44^ SXA is performed using an SXA phantom attached to a conventional digital mammography machine.^44^ The phantom is compressed to the same thickness as the breast. Data for this feasibility study was obtained using version 6.5 of SXA software.

The American College of Radiology's BI‐RADS system^45^ categorizes mammographic density into four classifications: (1) fatty, (2) scattered fibroglandular tissue, (3) heterogeneously dense, and (4) extremely dense. The BI‐RADS has been found to predict a four‐fold change in breast cancer risk between the first and fourth categories.^46^ Variability in compression during mammography and the reliance on trained readers to manually determine density reduces the sensitivity of BI‐RADS.^46^ Breast density data were collected at Marin County locations with digital mammography and were obtained from the SFMR through a cooperative agreement.

### 2.4 Saliva sample collection

Participants enrolled in the MWS were given the option to donate a saliva sample. Of them, 89% consented to donate saliva. Each one of the consented subjects was mailed a kit containing a collection tube and asked to provide an early morning saliva sample, before brushing teeth. Kits were mailed back to the Buck Institute for Research on Aging (Novato, CA) in pre‐addressed, postage‐paid envelopes for processing and storage. A total of 8700 specimens were received. The specimens were processed by separation into supernatant and a cellular component from which DNA was isolated using Invitrogen's PureLink Genomic DNA kits. Several potential testing laboratories were contacted to provide assays of the submitted saliva samples using mass spectrometry, the same methodology currently used for blood and saliva levels. The University of California‐Davis laboratory was the selected reference laboratory for the samples. The lab reported Cd results as (1) below minimal detectable levels (below 12 pg/L, the method detection limit), (2) detectable but with large confidence intervals (12–20 pg/L, the method reporting limit), and (3) reportable levels (above 20 pg/L). This study obtained 290 samples that were above 20 pg/L, the detectable limit. Under 20 pg/L is beyond the limit of the test. Conservatively, only 284 samples were analyzed that were 20 pg/L and above because the literature does not support any specific cut points. Therefore, the Cd saliva variable was analyzed as a continuous variable while excluding outliers.

### 2.5 Candidate selection

Only samples from white, non‐Hispanic females were selected to control for any potential confounding by race. Marin County has a highly homogeneous population and the great majority of subjects in the MWS were white non‐Hispanic. Subjects had to complete the MWS survey, have both a %FGV and BI‐RADS score, and donate two saliva specimens with at least 2 mL each of sample. All current cigarette smokers who met the other inclusion criteria (*n* = 48) were included. All other subjects, for a total of 400, were selected at random from among women meeting the other entry criteria. Conservatively, only 284 were selected for the analysis, after taking into account inclusion and exclusion criteria.

### 2.6 Statistical analysis

Saliva samples were grouped into batches for analysis with each containing 20 samples. Detectable levels were above 20 pg/L in 290 subjects, including outliers. Six outliers were removed before analysis. Univariate, bivariate, ANOVA, and logistic regression were performed on Cd categorized as above and below the mean value (55.7 [SD = 29.0]). Multivariate regression included age, BMI, smoking, and diet. All assumptions were met. Alpha level was assumed at 0.05. SPSS version 22 was used to analyze data.

## 3 RESULTS

Table 1 shows a subset of the MWS (*N* = 284), all current smokers (*n* = 33) and former smokers (*n* = 115) were tested for Cd using a saliva sample along with a random sample of non‐smokers (*n* = 136) from the MWS. Current smokers had a higher Cd mean (m = 61.4 pg/L) compared to non‐smokers (m = 54.5 pg/L) and former smokers (m = 55.3 pg/L) (Table 2), but these results were non‐significant. The study population characteristics are illustrated in Table 1. Cd was quantifiable and detectable in over 90% of the saliva samples. Cd saliva levels show a normal distribution with a mean of 55.7 and a standard deviation of 29.0 (See Figure S1). The levels varied from the lowest detectable level of 12 pg/L to 20 pg/L. Among the subjects of this study, 284 (m = 55.7, SD = 29.0) had salivary Cd levels above 20 pg/L. When using the BI‐RADS measurement, 123 (43.3%) participants had heterogeneous mammographic density, and 46 (16.2%) participants had extremely dense breasts. Moreover, there were 102 (35.9%) participants who had %FGV above 45%.

**TABLE 1:** Baseline Characteristics, *n* = 284.

Characteristic	*n* (%)
Age (years)	
≤55	153 (53.9)
>55	130 (45.8)
Body mass index	
Underweight/normal weight	207 (72.9)
Overweight/obese	74 (26.1)
Cigarette smoking status	
Never smoked	136 (47.9)
Former smoker	115 (40.5)
Current smoker	33 (11.6)
Energy intake	
Whole grain intake	
Yes	115 (40.5)
No	119 (41.9)
Vegetable intake	66 (23.2)
Yes	69 (23.8)
No	171 (60.2)
Cd, mean (SD)	55.7 (29.0)
BI‐RADS classification	
1‐Fatty	11 (3.9)
2‐Scattered fibroglandular tissue	104 (36.6)
3‐Heterogeneously dense	123 (43.3)
4‐Extremely dense	46 (16.2)
Breast density SXA (%)	
<25	86 (30.3)
25–<45	96 (33.8)
45+	102 (35.9)

*Note*: A subset of the MWS (*N* = 284), all current smokers (*n* = 33) and former smokers (*n* = 115) were tested for cadmium (Cd) using a saliva sample along with a random sample of non‐smokers (*n* = 136) from the MWS. Current smokers had a higher Cd mean (m = 61.4) compared to non‐smokers (m = 54.5) and former smokers (m = 55.3).

Abbreviations: BI‐RADS, Breast Imaging‐Reporting and Data System; MWS, Marin Women's Study; SXA, single x‐ray absorptiometry.

**TABLE 2:** Bivariate analysis with cadmium (Cd) (continuous) and potential confounders in subsection of population with detectable levels of Cd (*n* = 284).

	Saliva Cd levels (pg/L)	
	Mean (SD)	*t* (df), *p*‐value
Age		
≤55	57.0 (27.2)	0.863(281), *p* = 0.389
>55	54.0 (31.0)	
Body mass index		
Underweight/normal weight	55.5 (28.8)	0.329(279), *p* = 0.743
Overweight/obese	54.3 (26.4)	
Current smoker status		*F* (df); *p*‐value
Never smoker	54.5 (27.3)	0.762(2); *p* = 0.468
Former smoker	55.3 (29.1)	
Current smoker	61.4 (34.8)	
Whole grain intake		
Standard	55.6 (29.1)	0.813(232), *p* = 0.417
Not standard	58.6 (27.9)	
Vegetable intake		
Standard	58.2 (29.1)	−0.184(235), *p* = 0.854
Not standard	57.5 (28.9)	
2E. Edited with 20pg/L and under		*t*/*F* (df), *p*‐value
Age		
≤55	15.08 (3.34)	0.311 (71.46), *p* = 0.757
>55	14.88 (3.14)	
Body mass index		
Underweight/normal weight	15.27 (3.29)	1.75 (69.09), *p* = 0.085
Overweight/obese	14.18 (2.87)	
Current smoker status		
Never smoker	15.04 (3.38)	0.453 (2); *p* = 0.637
Former smoker	14.69 (3.02)	
Current smoker	15.57 (3.32)	
Whole grain intake		
Standard (high)	15.06 (3.27)	1.78 (51.20), *p* = 0.080
Not standard (low)	16.52 (2.73)	
Vegetable intake		
Standard (high)	15.25 (3.47)	0.657 (16.23), *p* = 0.521
Not standard (low)	15.98 (3.10)	

*Note*: This shows Cd levels shown for each population subsection. Each subsection was considered a potential confounder. A bivariate analysis between detectable saliva Cd levels (*n* = 284) and potential risk factors is reflected here. Participants who were ages 55 years and younger had a high Cd saliva mean of 57.0 compared to those older than 55 years (m = 54.0).

*Note*: It (2E) reflects that even when including the 20 and under, the results are the same as Table 2. Because the laboratory technicians were unable to verify the exact Cd levels when under 20 pg/L, we decided to exclude those samples.

A bivariate analysis between detectable saliva Cd levels (*n* = 284) and potential confounding variables is reflected in Table 2. Participants who were ages 55 years and younger had a high Cd saliva mean of 57.0 pg/L compared to those older than 55 years (m = 54.0 pg/L). Table 2 reflects that even when including Cd levels of 20 pg/L and under, the results are the same as Table 2. Table 3 shows that women with higher BI‐RADS classification (levels 3 and 4) and (mean, [SD] = 59.2 [31.7]) were statistically significantly more likely to have higher Cd saliva compared to those with lower BI‐RADS classification (levels 1 and 2) (mean, [SD] = 50.4 [23.6]) (t [df], *p* = −2.680 [280], *p* < 0.05).

**TABLE 3:** Univariate analysis with Cd versus SXA and Cd vs. Bi‐RADs, *n* = 284.

	Saliva Cd levels (pg/L)	
Variables	Mean (SD)	*F* (df); *p*‐value
Breast density SXA (%)		
<25	56.0 (28.9)	0.989 (2); *p* = 0.373
25‐ < 45	52.5 (29.0)	
45+	58.3 (29.0)	
BI‐RADS classification		
1‐Fatty	51.1 (23.4)	2.349 (3); *p* = 0.073
2‐Scattered fibroglandular tissue	50.3 (23.8)	
3‐Heterogeneously dense	60.3 (33.2)	
4‐Extremely dense	56.4 (27.1)	
BI‐RADS classification (2 levels)		*t* (df), *p*‐value
1‐2‐Fatty & scattered fibroglandular tissue	50.4 (23.6)	−2.680(280); *p* < 0.05
3‐4‐Heterogeneously dense & extremely dense	59.2 (31.7)	

*Note*: Women with higher BI‐RADS classification (levels 3 and 4) and (mean, [SD] = 59.2 [31.7]) were statistically significantly more likely to have higher Cd saliva compared to those with lower BI‐RADS classification (levels 1 and 2) (mean, [SD] = 50.4 [23.6]) (*t* [df], *p* = −2.680 (280); *p* < 0.05).

Abbreviations: BI‐RADS, Breast Imaging‐Reporting and Data System; SXA, single x‐ray absorptiometry.

Individuals with higher saliva Cd levels had an odds ratio of 1.34 for higher BI‐RADs after controlling for age, BMI, smoking, and diet (95% CI 0.75–2.39, *p* = 0.329) than individuals with lower saliva Cd levels, as seen in Table 4, which was not significant. While saliva Cd levels were not statistically significantly associated with BI‐RAD scores, the pilot data revealed that older age and higher BMI were associated with higher BI‐RAD scores after adjustment (*p* < 0.001). Table 4 shows the significance of age and BMI with BI‐RADS. Those who are younger than 55 have an odds ratio of 2.93 (*p* < 0.001) of having a higher BI‐RADS classification. Those who have a higher BMI have an odds ratio of 4.59 (*p* < 0.001) of having a higher BI‐RADS classification. Our univariate results also show that smoking is statistically significantly inversely associated with breast density (*p* < 0.05), as seen in Table S2.

**TABLE 4:** Logistic regression with BIRADS (2 levels), cadmium (Cd) above versus below mean and confounders, *n* = 284.

Variables	OR	95% CI	*p*‐value	Model fit
Constant for BI‐RADS classification	0.85			***R*** ^**2**^ **= 0.207**
Saliva Cd Levels (0 is low, 1 is high)	1.34	0.75–2.39	0.329	
Age	2.93	1.63–5.29	***p* < 0.001**	
BMI	4.59	2.34–9.03	***p* < 0.001**	
Smoking	1.75	0.95–3.23	0.075	
Veggie intake	0.98	0.52–1.86	0.954	

*Note*: Individuals with higher saliva Cd levels had odds ratio 1.34 after controlling for age, BMI, smoking and diet (95% CI 0.75–2.39, *p* = 0.329). While saliva Cd levels were not statistically significantly associated with BI‐RAD scores, the pilot data revealed that older age and higher BMI were associated with higher BI‐RAD scores after adjustment (*p* < 0.001). There is significance between age and BMI with BI‐RADS, as reflected by *p* < 0.05. Those who are younger than 55 have an odds ratio of 2.93 (*p* < 0.001) of having a higher BI‐RADS classification. Those who have a higher BMI have an odds ratio of 4.59 (*p* < 0.001) of having a higher BI‐RADS classification.

Abbreviations: BI‐RADS, Breast Imaging‐Reporting and Data System; BMI, body mass index.

## 4 DISCUSSION

The results of this pilot study indicated that while more work is needed to establish a more robust testing protocol before its usefulness can be qualified and quantified, meaningful salivary Cd measurement is possible. Further refinement of the process would be an appropriate next step to advance this work. Validating salivary Cd levels in comparison to blood and urine levels would also be necessary for establishing the usefulness of saliva for measuring Cd for use in large epidemiological studies with limited clinical access to patients.

In addition to examining the feasibility of obtaining a measure of salivary Cd, the present study allowed an examination of the feasibility of using saliva‐based Cd levels in association with breast density measures. In our study, we found that women with BI‐RADS classification of level 3 & 4 had statistically significantly higher detectable levels of Cd saliva compared to those with BI‐RADS classification of level 1 & 2. Since mammographic density is strongly associated with breast cancer, breast density, and breast cancer likely share common risk factors. Previous studies^47^, ^48^ have shown a relationship between Cd levels and breast density. Researchers showed that each two‐fold increase in urinary Cd was associated with 1.75 times the odds of having BI‐RADS classification of extremely dense in premenopausal women. There was a two‐fold increase in urinary Cd concentration associated with a statistically significant increase of 1.6% in mammographic density.^47^, ^48^ Positive associations between urinary Cd and fibro‐glandular tissue volume were shown in a group of nulliparous women,^47^, ^48^ in which a doubling of urinary Cd was associated with a 1.34‐fold change in the fibro‐glandular tissue volume.

Although these findings are encouraging, there are several limitations to note. The small sample size in the present study limited our ability to demonstrate statistical significance despite suggestions of a positive association between salivary Cd and breast density, consistent with previous studies that have investigated associations of Cd exposure, breast density, and breast cancer.^49^, ^50^, ^51^, ^52^, ^53^ Additionally, many of our demographic and behavioral variables were self‐reported and we recognize those limitations. However, because participants are likely unaware of their Cd (pg/L) status, this bias is likely non‐differential and caused our measures of association to bias toward the null. Our study found that age and BMI are statistically significant with BI‐RADS and are trending toward significance in saliva Cd and smoking. Furthermore, salivary quantification of Cd, though not considered the gold standard for diagnostic or screening methods of Cd levels, has the potential for cost‐effective utilization and validity, as previously considered by other studies.^50^

## 5 CONCLUSION

In conclusion, we found that saliva‐based sampling for Cd (pg/L) has the potential as a bio‐sample to measure Cd (pg/L) and should be considered for larger epidemiological studies where physical access to patients is limited or challenging. While these results are not statistically significant or ground‐breaking evidence for an association between breast density and salivary Cd level (pg/L), the introduction of a method of gathering biosamples for cancer risk screening that is less invasive, more convenient, and cost‐effective means is promising. Evidence in what is currently available in the literature points to Cd as a carcinogen, contributing to cancer development. Therefore, further research and investigations should move toward the determination of salivary Cd and its usefulness in breast cancer screening.

### 5.1 Clinical practice points

Existing evidence available in the literature included the identification of risk factors for breast cancer development or predisposition. Such risk factors include intrinsic and unmodifiable genetic composition. Modifiable risk factors include smoking status, environmental exposures to known carcinogens, and dietary intake of foods that are scientifically accepted as potentially carcinogenic. Early detection of breast cancer has shown efficacy for the prolongation of life and preserving high quality of life. The findings of this feasibility study using salivary Cd levels (pg/L) as predictors of breast cancer disposition include the potential for a very cost‐effective and convenient method of breast cancer risk factor screening. The findings also add to the already existing evidence that breast density plays a critical role in the pathogenesis of breast cancer development. There is an ongoing need in clinical practice and breast cancer research to advance the search for methodologies that are not only cost‐effective, appropriate, and efficient but are also convenient for the target population. A screening risk factor tool with these elements should remove barriers in clinical care.

## AUTHOR CONTRIBUTIONS

**Michaela George:** Formal analysis (lead); supervision (equal); writing – original draft (lead); writing – review and editing (equal). **Shayne Paff:** Writing – original draft (equal); writing – review and editing (equal). **Jenyse Rojo:** Formal analysis (equal); writing – original draft (equal). **Mark Powell:** Data curation (supporting); project administration (equal); resources (equal); writing – review and editing (equal). **Christopher Benz:** Conceptualization (equal); data curation (equal); writing – review and editing (equal). **Karl Pope:** Formal analysis (lead); writing – review and editing (equal). **Karla Kerlikowske:** Conceptualization (equal); data curation (lead); investigation (equal); writing – review and editing (equal). **John Shepard:** Data curation (lead); investigation (equal); writing – review and editing (equal). **Matthew Willis:** Supervision (equal); writing – review and editing (equal). **Rochelle Ereman:** Conceptualization (lead); investigation (equal); methodology (lead); visualization (lead); writing – original draft (supporting). **LeeAnn Prebil:** Conceptualization (equal); investigation (equal); methodology (equal); project administration (equal); visualization (equal); writing – original draft (equal).

## CONFLICT OF INTEREST STATEMENT

The authors have declared that there are no conflicts of interest that exist.

## Supporting information


Data S1:

Data S2:

## Data Availability

The data that support the findings of this study are available on request from the corresponding author first author. The data are not publicly available due to privacy or ethical restrictions. However, if de‐identified data is requested, the data that support the findings of this study may be available from the first author upon reasonable request.
