# Supplementary material for: Assessment of salivary cadmium levels and breast density in the Marin Women's Study
Source: Cancer Med. 2024 Feb 1;13(2):e6973. doi: 10.1002/cam4.6973 (PMC10831917; doi:10.1002/cam4.6973)
Supplement: Supplementary file 1 — Data S1: [file CAM4-13-e6973-s001.docx]

**Supplemental Materials**

**Figure S1C. Relevant Questions from Questionnaire in the Marin Women’s Study**


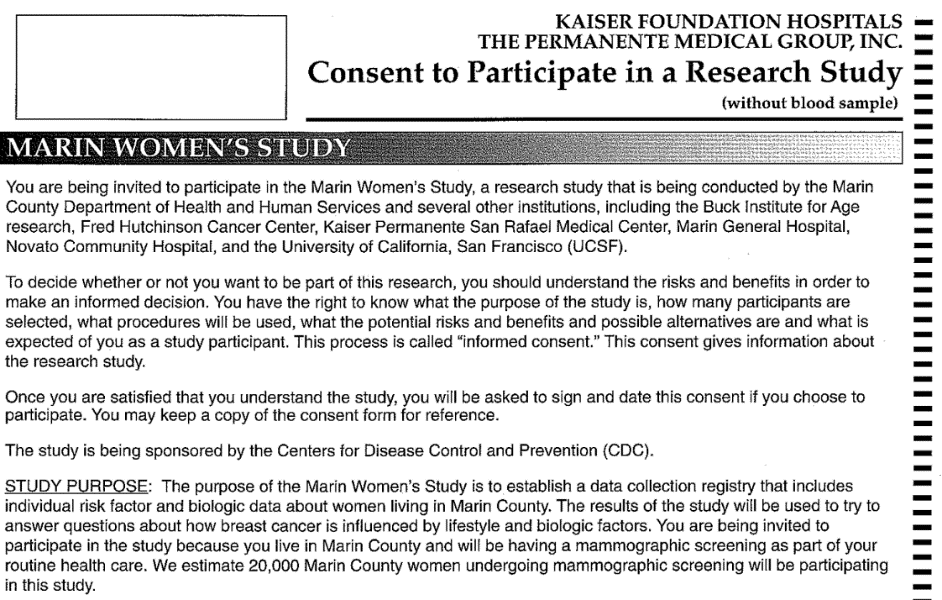


**
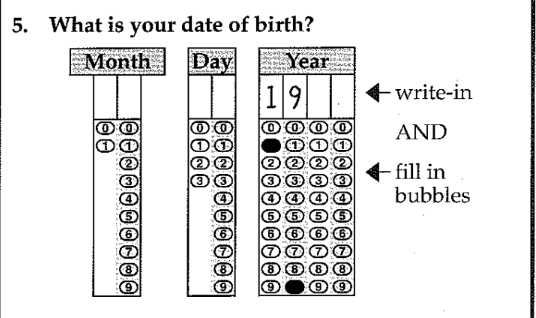
**

**
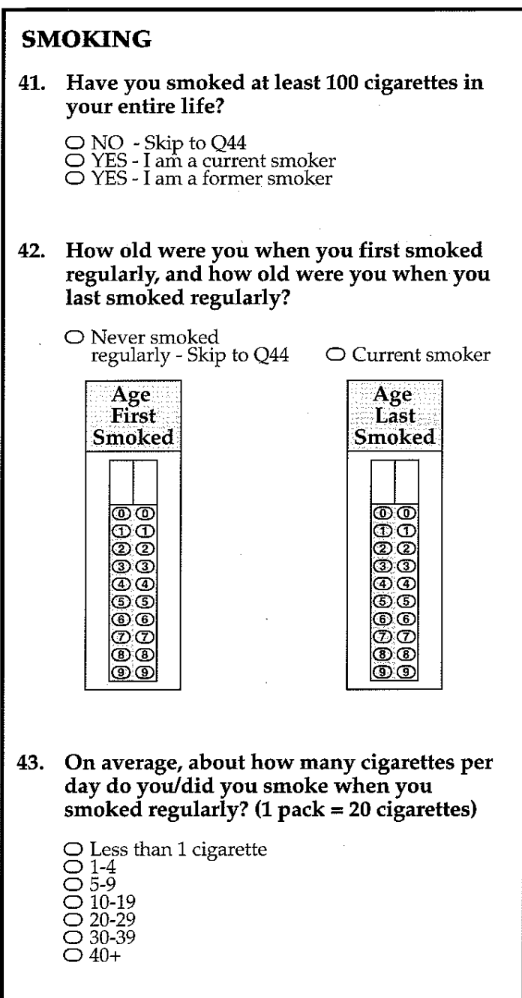
**

**
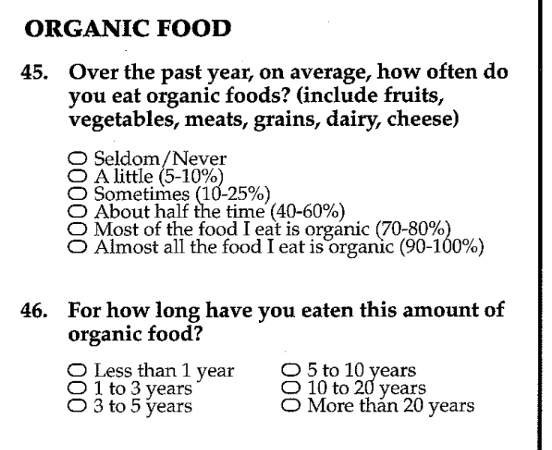
**

**
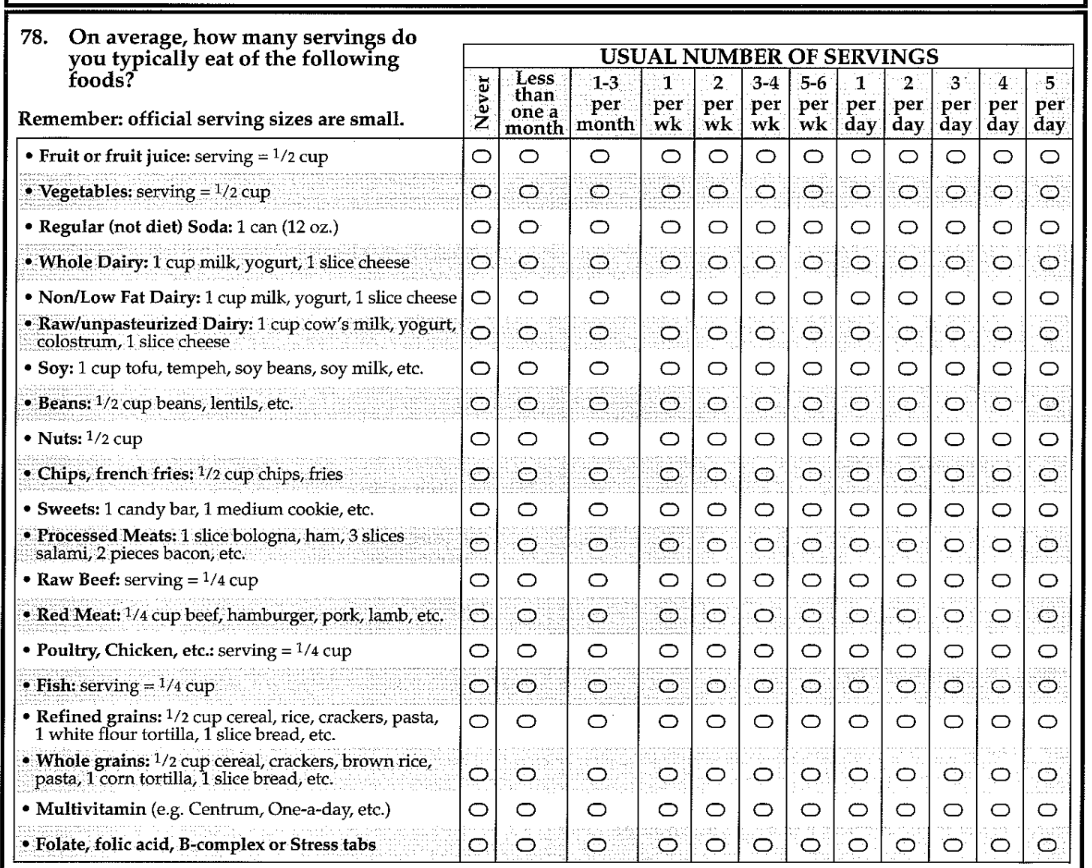
**

**Figure S1A. Distribution of Cd saliva levels pg/L, n=284**

**
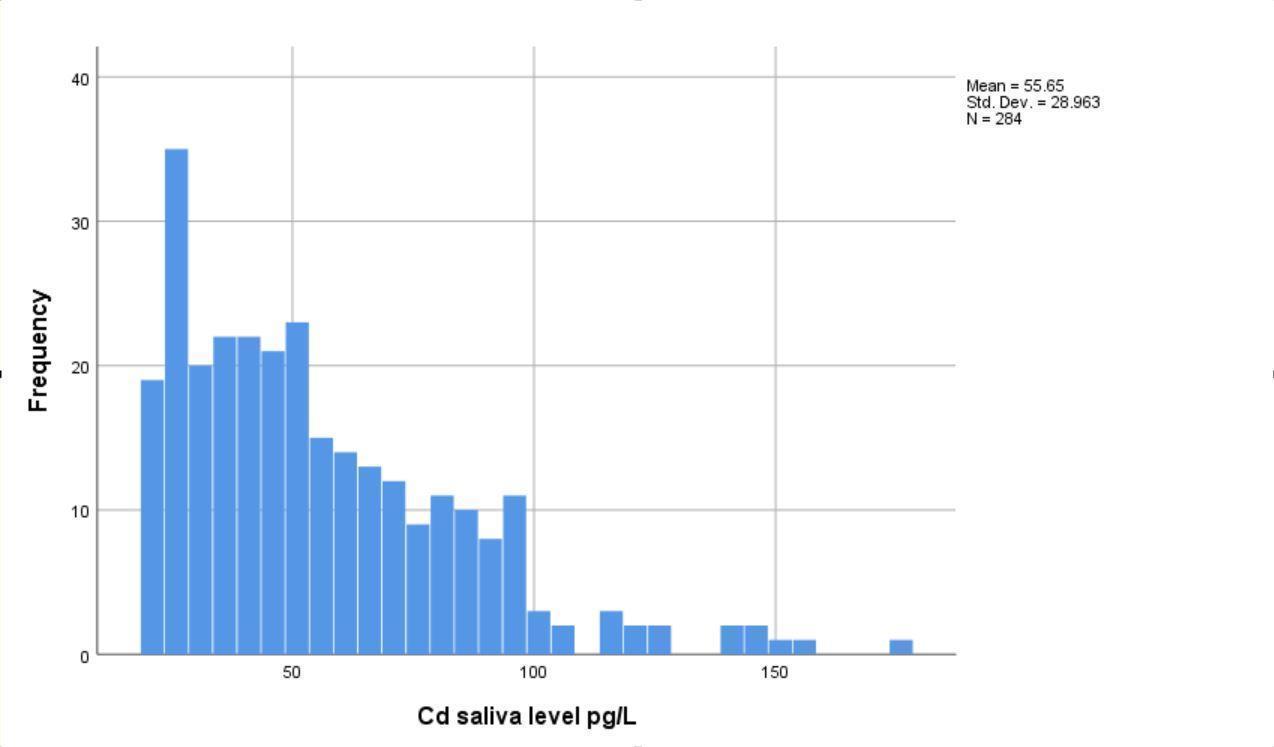
**

**Normal distribution of Cd, data from all participants in the study.**

**Figure S1B. Distribution of Cd saliva levels pg/L, n=400**

**
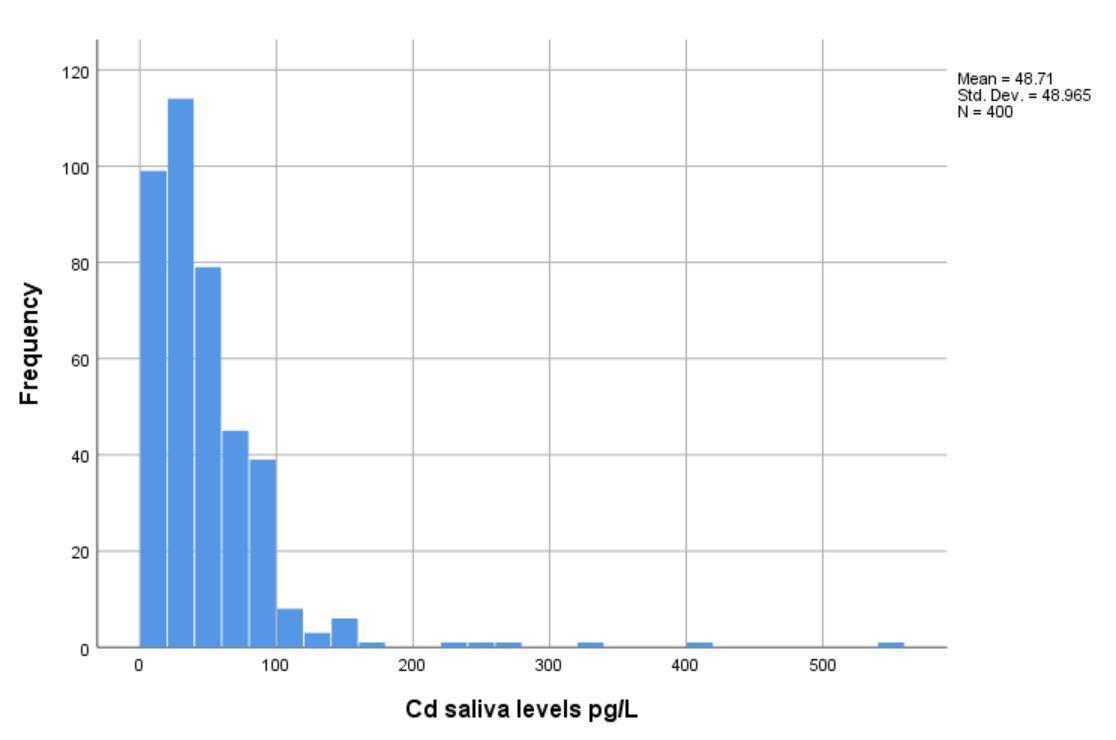
**

**Normal distribution of Cd, data from all participants in the study.**

**Table S1: Baseline Characteristics, n=400**

| **Characteristic** | **n(%)** |
| --- | --- |
| **Age (years)** |  |
| ≤ 55 | 196 (49.0) |
| > 55 | 203 (50.7) |
| **Body Mass Index (BMI)** |  |
| Underweight/ Normal Weight | 288 (72.0) |
| Overweight/Obese | 109 (27.3) |
| **Cigarette smoking status** |  |
| Never smoked | 185 (46.3) |
| Current smoker | 48 (23.0) |
| Former smoker | 167 (41.8) |
| **Energy intake (kcal/day)** |  |
| **Whole grain intake (½ cup/day)** |  |
| Yes | 143 (35.8) |
| No | 150 (37.5) |
| **Vegetable intake (½ cup/day)** |  |
| Yes | 216 (54.0) |
| No | 81 (20.3) |
| **Saliva Cd levels (pg/L)** |  |
| < 12 | 32 (8.0) |
| 12-20 | 78 (19.5) |
| > 20 | 290 (72.5) |
| **Cd is in the >20, mean (SD)** | 48.7 (49.0) |
| **BI-RADS classification** |  |
| 1- Fatty | 15 (3.8) |
| 2- Scattered fibroglandular tissue | 145 (36.3) |
| 3- Heterogeneously dense | 179 (44.8) |
| 4- Extremely dense | 61 (15.3) |
| **Breast density SXA (%)** |  |
| <25 | 125 (31.3) |
| 25-<45 | 142 (35.5) |
| 45+ | 133 (33.3) |

*Table S1* reflects the characteristics of participant subgroups out on sample size n=400.
