# Supplementary material for: Assessment of salivary cadmium levels and breast density in the Marin Women's Study
Source: Cancer Med. 2024 Feb 1;13(2):e6973. doi: 10.1002/cam4.6973 (PMC10831917; doi:10.1002/cam4.6973)
Supplement: Supplementary file 2 — Data S2: [file CAM4-13-e6973-s002.docx]

**Table S2. Linear Regression analysis with Cd and SXA, n=284**

| **Variables** | **Beta** | **SE** | **p-value** | **Model Fit** |
| --- | --- | --- | --- | --- |
| **Constant for Breast Density SXA (%)** | 2.164 |  |  | **R^2^= 0.386** |
| **Saliva Cd Levels (pg/L)** | 0.00 | 0.00 | 0.170 |  |
| **Age** | -0.68 | 0.09 | 0.000 |  |
| **BMI** | -0.70 | 0.10 | 0.000 |  |
| **Smoking** | -0.39 | 0.13 | 0.003 |  |
| **Veggie intake** | -0.05 | 0.09 | 0.578 |  |
